# Atopic dermatitis, cutaneous steroids and cataracts in children: two case reports

**DOI:** 10.1186/1752-1947-2-124

**Published:** 2008-04-28

**Authors:** Andrew Tatham

**Affiliations:** 1 Leicester Royal Infirmary, Infirmary Square, Leicester LE1 5WW, UK

## Abstract

**Introduction:**

Atopic dermatitis is a chronic, pruritic, eczematous skin disease mediated through an immediate (type I) hypersensitivity reaction. Posterior sub-capsular cataracts are a recognised complication of atopic dermatitis in adults; however they are rare in children. The management of atopic dermatitis is based on the exclusion of allergens, the use of emollients, and on topical corticosteroids for disease exacerbations. Cataracts may be due to atopic dermatitis but may also occur secondary to the use of corticosteroids.

**Case presentation:**

We describe two children with atopic dermatitis, treated with cutaneous corticosteroids, both of whom were diagnosed with bilateral posterior sub-capsular cataracts.

**Conclusion:**

These cases demonstrate that atopic dermatitis and topical corticosteroids may be associated with cataracts in children as well as adults. The cause of cataracts in atopic dermatitis is not known, however, it has been suggested that habitual tapping and rubbing of the face may play a role. Care needs to be taken when prescribing corticosteroids. Inadequate treatment of atopic dermatitis may lead to other ocular complications such as keratitis and permanent visual loss.

## Introduction

Atopic dermatitis (AD) is a chronic, pruritic, eczematous skin disease mediated through an immediate (type I) hypersensitivity reaction. It primarily affects the flexural surfaces and lesions exhibit a red, elevated, scaly and often excoriated appearance. AD is typically manifest in infants aged 1 to 6 months and 90% of eventual sufferers have had their first outbreak by age 5 years. Ocular complications of AD in adults include blepharitis, keratoconjunctivitis, keratoconus, uveitis, sub-capsular cataract and retinal detachment. Cataracts secondary to AD may occur in 25 to 50% of adults but are rare in adolescents and young adults [1]. The most common ocular finding in children is a papillofollicular conjunctivitis [1]. Two main types of cataract are seen in patients with AD, an anterior sub-capsular plaque and anterior and posterior sub-capsular opacities.

The management of AD is based on the exclusion of allergens, the use of emollients and on topical corticosteroids for disease exacerbations. Cataracts may be due to AD but may also occur secondary to the use of corticosteroids. The cataract associated with corticosteroids tends to be posterior sub-capsular. Ocular complications of corticosteroids may occur following intravenous, oral, inhaled or ocular administration. Although corticosteroids are commonly used in the treatment of dermatological diseases there are few reports of cataracts occurring following the use of cutaneous corticosteroids [1,2].

## Case presentation

We report two cases of children with AD who developed posterior sub-capsular cataracts.

### Patient 1

**Figure 1:**
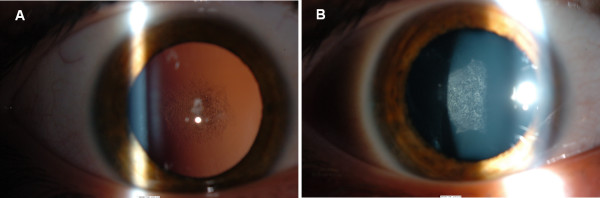
Posterior sub-capsular cataract in the right (A) and left eye (B) in patient 1.

A 13-year-old boy presented to the paediatric ophthalmology clinic with a 2-year history of progressive blurring of vision in the right eye. He had a past history of AD, which was diagnosed at age 2 years. He had required regular hydrocortisone 1% cream to control his symptoms. Typically he was using a 30 g tube every 3 to 4 weeks on his arms and face. On examination, his vision was 2/60 in the right eye and 6/5 in the left eye. He was noted to have a posterior sub-capsular opacity of the right lens (Figure 1A). There was no family history of note and his parents had normal ocular examinations. A cataract extraction and intra-ocular lens implantation was performed but unfortunately his surgery was complicated by staphylococcal endophthalmitis which presented on day 5 postoperatively. Despite this major setback, he made a good recovery and his vision 3 years later was 6/9 in the right eye.

Over the following 2 years, he developed a posterior sub-capsular cataract in his left eye (Figure 1B) and his vision reduced to light perception only. Cataract extraction and intra-ocular lens insertion was successfully performed on his left eye and his vision in now 6/6 in this eye. He continues to require 0.5% hydrocortisone to his face and 1% hydrocortisone on his arms.

### Patient 2

**Figure 2:**
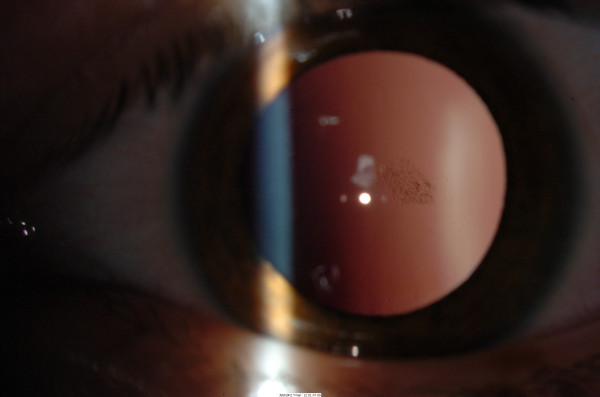
Posterior sub-capsular cataract in the right eye in patient 2.

An 8-year-old boy presented to the paediatric ophthalmology clinic complaining of gradual onset of blurred vision in both eyes. On examination, his vision was 6/36 in the right eye and 6/9 in the left eye, with no improvement with pinhole. On slit-lamp examination, he was noted to have bilateral posterior sub-capsular cataract, which was worse in the right eye (Figure 2). He was tested for spectacles with which his vision improved to 6/9 in the right eye and 6/6 in the left. The child had being using topical steroids for the previous 2 years after being diagnosed with widespread AD of the face, neck, trunk and limbs. Given his good corrected visual acuity and following discussion with his parents, cataract extraction has not yet been performed.

## Discussion

The cataracts in these patients may have been due to the underlying disease, the treatment, or a combination of both. Cataracts secondary to AD are rare in children. In a series of 59 children, there was just one case of cataract [1]. Why cataracts develop in patients with AD is not known, however, habitual tapping and rubbing of the face, a common problem in pruritic conditions, may play a role [3]. Indeed, the presence of facial skin lesions in AD correlates with progression of the cataract [3]. An alternative hypothesis suggests that the cataract is secondary to compromise of the blood-aqueous barrier. Patients with AD have been found to have higher levels of protein flare in the aqueous humour than controls [4]. Posterior sub-capsular cataracts may also be caused by corticosteroids used in the treatment of AD.

The association between systemic corticosteroids and posterior sub-capsular cataracts was first noted by Black et al. [5]. Subsequent studies have shown that corticosteroid-induced cataracts may develop following even small doses of steroids, particularly in children. Posterior sub-capsular cataract may occur at a faster rate and lower dosage in children [6]. In addition to systemic steroids, cataracts have also been associated with ocular topical steroids, inhaled steroids and topical steroid creams [7,8]. When steroids are applied topically to the skin, the degree of systemic absorption depends on factors such as drug potency, the duration of application and whether the skin is thin or damaged. Even low potency steroid creams applied to the eyelids may result in increased intra-ocular pressure and cataract [9].

The mechanism of corticosteroid-induced cataract is not known but may be due to osmotic imbalance, oxidative damage or disrupted lens growth factors [2]. The osmotic theory suggests that corticosteroids interfere with the ionic composition of the lens. The oxidative theory proposes that corticosteroids inhibit the normal mechanisms that protect the lens from oxidative stress. Another theory of cataract formation proposes that steroids influence lens-related growth factors. Normal lens growth is mediated by growth factors such as fibroblast growth factor-2 present in the aqueous and vitreous humour [2]. Corticosteroids may influence lens epithelial cell behaviour by interfering with the normal production of growth factors. This effect may result in undifferentiated anterior epithelial cells migrating and accumulating at the posterior pole forming a posterior sub-capsular cataract [2].

Cataract extraction in children with atopic cataract can produce excellent visual results, however, it is important to consider the presence of a coexisting retinal detachment. Retinal detachment has been reported in 8% of patients with AD and in one series, 25% of eyes with atopic cataract had retinal breaks or detachment noted pre-operatively [10]. A rapidly progressing cataract may mask the presence of a shallow retinal detachment and an unrecognised retinal detachment can cause a mild cataract to progress faster. B-scan ultrasonography is a useful investigation to evaluate the anatomy of the retina in these eyes. Retinal detachment may also be associated with panuveitis or hypotony [11].

## Conclusion

Corticosteroids are known to cause posterior sub-capsular cataract by most routes of administration. After diabetes, myopia and glaucoma, steroid use is the fourth leading risk factor for secondary cataract and accounts for 4.7% of all cataract extractions [2]. Care needs to be taken when prescribing corticosteroids, particularly in children who have a lower susceptibility to side effects. Corticosteroids are frequently required to adequately treat AD, to limit pruritus and prevent complications such as keratitis that can lead to permanent visual loss. Exacerbations of AD need to be treated aggressively. Many patients and parents have negative perceptions regarding the use of steroids, which may lead to inadequate treatment of the skin disease and increased eye rubbing. The increasing use of alternative specific immunosuppressants that lack the side effect profile of corticosteroids may reduce the incidence of cataracts in these patients.

## Abbreviations

AD: atopic dermatitis.

## Competing interests

The author declares that they have no competing interests.

## Authors' contributions

AT examined the patients, conducted the literature review and wrote the manuscript.

## Consent

Written informed consent was obtained from the patients' parents for publication of this case report and accompanying images. A copy of the written consent is available for review by the Editor-in-Chief of this journal.
